# Models of community hospitals and state of research in high-income countries: a scoping review

**DOI:** 10.3389/fpubh.2024.1507729

**Published:** 2025-01-23

**Authors:** Min Hui Tan, Sharna Si Ying Seah, Xin Yi Seah, Simone Teo, Jeremy Leow, Lian Leng Low

**Affiliations:** ^1^SingHealth Community Hospitals, Singapore, Singapore; ^2^Singapore General Hospital, Singapore, Singapore; ^3^SingHealth Duke-NUS Family Medicine Academic Clinical Programme, Singapore, Singapore

**Keywords:** post-acute care, models of care, community hospitals, research, scoping review

## Abstract

**Introduction:**

Existing literature have not reviewed the growing spectrum of care models in Community Hospitals (CH) along with the scope of research. We fill this gap by reviewing CHs models in high-income countries.

**Methods:**

We conducted a scoping review according to Arksey & O’Malley’s framework. We searched for articles published between January 2016 to April 2024 in EMBASE, PubMed, and Scopus. Additional studies were identified through snowballing.

**Results:**

470 studies were included in the review. CHs models in 22 countries were categorized based on healthcare services provided and target patient populations. CHs in 18 countries were found to provide COVID-19 services. CHs in eight countries primarily provide post-acute and rehabilitative services. 40 articles were extracted to synthesize research themes in CHs providing post-acute care. Majority focused on assessing the healthcare needs of patient populations. Other domains include program efficacy, research and educational needs of staff, clinical guidelines reviews, and the community’s role in supporting CHs.

**Conclusion:**

CHs evolve to meet changing healthcare needs and understanding the state of CHs research would inform potential research directions. Future studies could explore the relationship between post-acute settings and the community, and strategies to enhance staff capability and address barriers to conducting research in post-acute settings.

## Introduction

On a global scale, there has been a surge in demand for healthcare services to meet the needs of ageing populations, which involves organizing healthcare services around patients’ needs to produce better outcomes (1, 2). Furthermore, one of the United Nations’ goals for the 2030 Agenda for Sustainable Development is to improve physical and mental health by attaining universal health coverage and access to high quality healthcare (3). In line with this goal; care models – defined as the provision of healthcare services by a healthcare institution (4) – would need to adapt to meet the populations’ evolving needs. This is through improvement of healthcare infrastructure and workforce capacity.

Community Hospitals (CHs) typically facilitate the transition of patients from acute hospitals to the community, traditionally cater to rural populations, and have multidisciplinary teams (5).

To date, CH models vary according to their population needs (6–8). While a previous scoping review of CHs identified the scope of service provision in CHs (5), it was only conducted in 10 high-income countries in Western and rural settings. The omission of CHs in Asian contexts does not account for the burgeoning role of CHs in these countries. With the increasing importance of CHs, there is a need to develop a research agenda to enhance service delivery. Therefore, we aim to update a previous review by Winpenny and colleagues (5) by performing a scoping review to: (i) consolidate care models in CHs in *both* Asian and Western high-income countries and (ii) examine the research areas and gaps in CHs focusing on post-acute care. This will enable healthcare administrators to gain a comprehensive overview of the various CH models and research directions that can further evidence-based practice.

## Methods

Our scoping review was guided by scoping review methodological frameworks by Levac *et al.* (9), Arksey and O’Malley (10), and the PRISMA-ScR checklist (11). The protocol for this scoping review is registered on 8 June 2024 on Open Science Framework (Available online: https://osf.io/j74fq/?view_only=04582d9ab97e4f5e869514edbb176d31). The data that support the findings of this study are available from the corresponding author upon reasonable request.

### Eligibility criteria and information sources

We performed a systematic search strategy for articles published between January 2016 to April 2024 in three databases – EMBASE, PubMed, and Scopus. We excluded studies that were conducted in a home setting and in a non-high-income country. According to the World Bank (12), high-income countries have a Gross National Income (GNI) per capita of more than US$13,846 in 2024. Institutional review board approval was not obtained as human subjects were not involved. The inclusion and exclusion criteria are recorded in Table 1.

**Table 1:** Inclusion and exclusion criteria for selection of studies to include for identifying models of community hospitals.

Criterion	Inclusion	Exclusion
Publication status	Already published	Not published
Period	January 2016, until April 2024	Before January 2016
Language	English	All other non-English languages
Study setting	Meets all of the following criteria: provides beds for inpatient stay is led by community-based health professionals provides a range of services to a local community	Does not meet the following criteria: provides beds for inpatient stay is led by community-based health professionals provides a range of services to a local community
Outcomes	A description of the nature and scope of delivery models/ services OR name of hospital is provided	Does not describe the nature and scope of delivery models/ services AND does not provide the name of the individual hospitals
Country	High-income countries (GNI per capita > US$13,846) with comparable healthcare systems	Non-high-income countries (GNI per capita ≤ US$13,846)

### Search strategy

The search strategy comprised of the search terms: “community hospitals” and “cottage hospitals”. The concepts included in the search strategy were adapted from a scoping review of CHs in high-income countries (5). The search strings were developed through iterative discussions within the team and have been presented in Table 2. Additional studies were identified through backward and forward snowballing.

**Table 2:** Search strategies and sources for EMBASE, PubMed, and Scopus.

Sources	Date	Search strategy
EMBASE	30 April 2024	(‘community hospital’:ab,ti OR ‘community hospitals’:ab,ti OR ‘cottage hospital’:ab,ti OR ‘cottage hospitals’:ab,ti OR ‘gp beds’:ab,ti OR ‘intermediate care’:ab,ti) AND [2016–2024]/py AND (‘article’/it OR ‘article in press’/it OR ‘conference paper’/it)
PubMed	30 April 2024	“Hospitals, Community”[Mesh] OR “Hospitals, Group Practice”[Mesh] OR “Hospitals, Rural”[Mesh] OR (“Hospitals”[Mesh] AND “Family Practice”[Mesh]) OR (“Hospitals”[Mesh] AND “Rural Health Services”[Mesh]) OR (“Family Practice”[Mesh] AND “Hospital-Physician Relations”[Mesh]) OR “Hospital Bed Capacity, under 100”[Mesh] OR (“Family Practice”[Mesh] AND “Bed Occupancy”[Mesh]) OR “Intermediate care facilities”[Mesh] OR “cottage hospital”[All Fields] OR “cottage hospitals”[All Fields] OR “community hospital”[All Fields] OR “community hospitals”[All Fields] OR (“gp”[All Fields] AND (“beds”[MeSH Terms] OR “beds”[All Fields] OR “bed”[All Fields])) OR “gp beds”[All Fields] OR “general practitioner hospital”[All Fields] OR “general practitioner hospitals”[All Fields] OR ((“community”[All Fields] OR “rural”[All Fields]) AND “hospitals, maternity”[MeSH Terms]) OR “intermediate care” [All Fields] NOT (“Africa”[Mesh] OR “Africa, Western”[Mesh] OR “Africa, Central”[Mesh] OR “South Africa”[Mesh] OR “Africa, Southern”[Mesh] OR “Africa, Northern”[Mesh] OR “India”[Mesh] OR “China”[Mesh] OR “South America”[Mesh] OR “Developing Countries”[Mesh])
Scopus	30 April 2024	TITLE-ABS-KEY (“community hospitals” OR “community hospital”) OR TITLE-ABS-KEY (“cottage hospital” OR “cottage hospitals”) OR TITLE-ABS-KEY (“intermediate care”) AND (EXCLUDE (AFFILCOUNTRY,"China”) OR EXCLUDE (AFFILCOUNTRY,"Thailand”)) AND (LIMIT-TO (DOCTYPE,"ar”)) AND (LIMIT-TO (PUBYEAR,2024) OR LIMIT-TO (PUBYEAR,2023) OR LIMIT-TO (PUBYEAR,2022) OR LIMIT-TO (PUBYEAR,2021) OR LIMIT-TO (PUBYEAR,2020) OR LIMIT-TO (PUBYEAR,2019) OR LIMIT-TO (PUBYEAR,2018) OR LIMIT-TO (PUBYEAR,2017) OR LIMIT-TO (PUBYEAR,2016)) AND (LIMIT-TO (LANGUAGE,"English”))

### Selection of sources of evidence, data charting process, and data items

The citations retrieved were exported into Zotero. Duplicated citations were removed before screening. First, ET and XY performed the initial pilot exercise by screening the first 500 records (based on title and abstract), selecting articles that met the inclusion criteria. After which, ET, ST, and JL independently screened the remaining titles and abstracts of all articles from the three databases. Unresolved disagreements were independently arbitrated by a fifth independent reviewer (SS).

The charting of data from the included articles was performed using a standardized data collection sheet according to the following categories: authors, study title, year published, country studied, overview of the study, target patient population, type of care provided, and type of research conducted in CHs providing mainly post-acute care.

Due to the diversity of included studies, the broad scope of our research questions, and the scoping nature of this review, we did not proceed with a formal quality assessment. For example, only nine cohort studies and three randomized controlled trials were included. However, the study team made a note of the nature of the evidence and highlighted any concerns regarding the quality of the studies during the data extraction process.

### Models of CHs

The services rendered by CHs were inferred from either: (i) the background information of the article, or (ii) the respective hospital’s official website. We built our results upon the exposition of the types of services provided in Winpenny *et al*.’s study (5).

### Research conducted in CH settings which provide only post-acute care

We decided to focus on research conducted in CHs providing mainly post-acute care to patients – similar to the model of care which was first developed in the UK (6, 13). Full texts were then extracted and analyzed for key themes.

### Summary and synthesis of results

#### CH models of care

**Figure 1:**
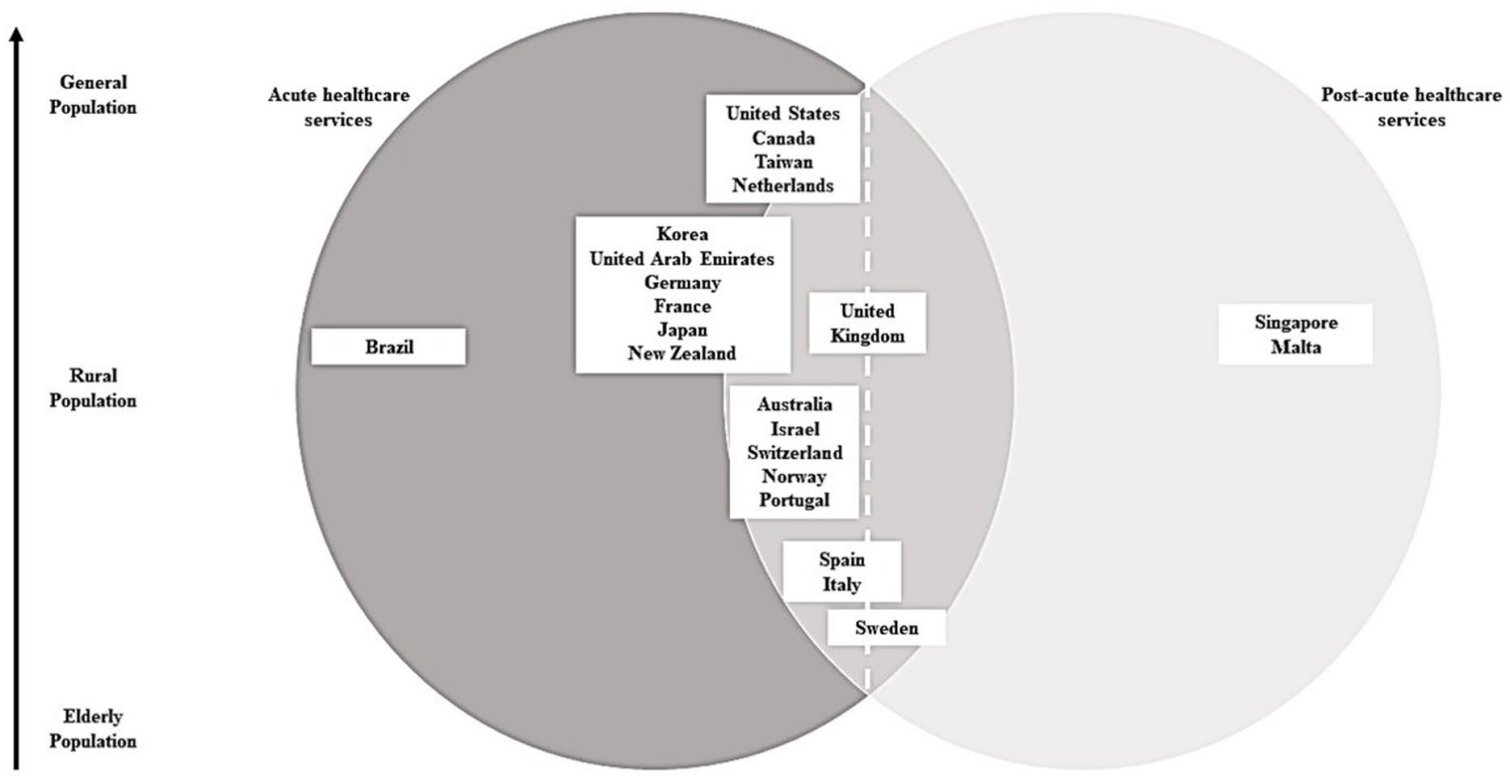
Venn diagram depicting types of services provided by community hospitals in high-income countries.

ET, ST, and JL retrieved the CHs’ definitions and independently classified them based on the healthcare services provided and target patient population before organizing them in a Venn diagram (Figure 1).

#### Research conducted in CHs providing only post-acute care

We focused on research topics that were carried out in post-acute care settings as they bear resemblance to the model of care that was first developed in the UK (6, 13). Eligible articles were coded independently by ET and XY through inductive and deductive coding methods. The predefined set of codes included: research aim, methodological approach, target population, findings, and study limitations. During the inductive coding process, new codes were established based on the data and constant comparative analysis was conducted after a first round of coding was completed to derive key thematic categories. The key research themes in CHs and its order of dominance was identified based on the number of references. Any disagreements in coding were reconciled by both coders.

## Results

**Figure 2:**
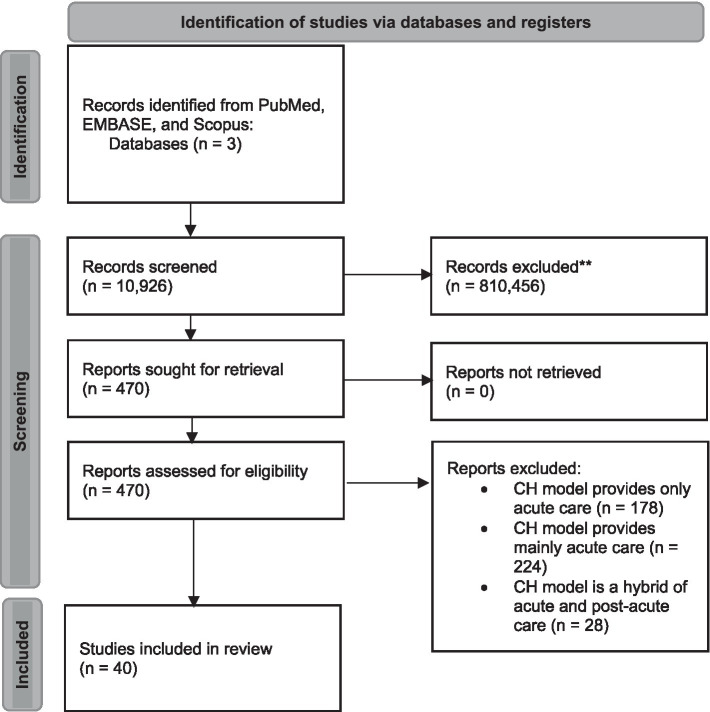
The PRISMA flow chart. PRISMA, preferred reporting items for systematic reviews and meta-analysis.

A flowchart detailing the inclusion of articles can be found in Figure 2.

### Models of CHs in different countries

To identify the CH models of different countries, we retrieved a total of 10,926 citations following the removal of duplicates. Upon screening of titles and abstracts, 470 papers were eligible for inclusion to map CH models (Figure 2) in 22 high-income countries. Table 3 summarizes the models and number of studies conducted in high-income countries.

**Table 3:** Origins of studies included for reviewing models of community hospitals (*n* = 470) and models of care in different countries (*n* = 22).

Variables	*n* (%)
Continent and country of study	
*North and South America*	
United States	249 (53.0%)
Canada*	34 (7.2%)
Brazil	1 (0.2%)
*Asia*	
Japan	54 (11.5%)
Singapore	21 (4.5%)
Taiwan	9 (1.9%)
Korea	5 (1.1%)
United Arab Emirates	2 (0.4%)
Israel	3 (0.6%)
*Europe*	
United Kingdom*	23 (4.9%)
Netherlands*	11 (2.3%)
Italy	9 (1.9%)
Spain	8 (1.7%)
Germany	7 (1.5%)
Switzerland	6 (1.3%)
Norway*	4 (0.9%)
Sweden	3 (0.6%)
France	3 (0.6%)
Malta	1 (0.2%)
Portugal	1 (0.2%)
*Oceania*	
Australia*	8 (1.7%)
New Zealand*	7 (1.5%)
*Others*	
Multiple countries	1 (0.2%)
CH care model	
Post-acute	39 (8.3%)
Acute	178 (37.9%)
Hybrid (emphasis on acute)	224 (47.7%)
Hybrid (emphasis on post-acute)	1 (0.2%)
Hybrid (equal emphasis on acute and post-acute)	28 (6.0%)
Provision of COVID-19 healthcare services	
Yes	152 (32.3%)
No	258 (54.9%)
Specific CH not mentioned	60 (12.8%)

Countries marked with an asterisk * refer to countries that were originally included in Winpenny and colleagues’ study (5).

The CHs also differed in terms of COVID-19-related services provided based on the context they operate in. In addition to the countries included in Winpenny’s study (5), we included several others, such as: The United States, Brazil, Japan, Taiwan, United Arab Emirates, Israel, Italy, Spain, Germany, Switzerland, Sweden, France, Malta, and Portugal.

The countries were categorized according to the types of healthcare services provided and their target patient population indicated within the parentheses (Figure 1). Countries in the intersection provide some level of acute and post-acute care services. Within the intersection, countries that fall on the left side of the dotted line offer mainly acute services, and some post-acute services; countries on the right of the dotted line provide mainly post-acute services, and some acute services.

In Figure 1, three countries (13.6%) mainly provide post-acute services, 16 countries (72.8%) offer primarily acute services, and three countries (13.6%) fall at the intersection - providing both post-acute and acute care.

### Types of healthcare services

There is no uniform CH model of care. Each CH has its own combination of services to cater to different needs of its patient population. For example, due to a rapidly ageing populace, some countries organize their CH models around sub-acute care, providing services such as: post- and sub-acute care, rehabilitative services, and, in some cases, palliative or end-of-life services (14). Countries with CH models that mainly focus on sub-acute care are the Singapore, Italy, Malta, and Sweden.

On one hand, some countries’ CHs are geared towards providing acute care, which comprises of emergency services, trauma care, surgical services, critical care, and urgent care (15). Countries with CH models that focus on acute care are Brazil, the United States, Canada, Japan, Taiwan, Netherlands, Korea, United Arab Emirates, Germany, France, Israel, Switzerland, and Norway.

Conversely, some countries’ CHs provide a hybrid model of care, such as the UK and Spain.

### Patient populations

Depending on local context, CHs cater to different populations, such as older adults, rural populations, to the general population. Some CHs within the same country may serve more than one type of patient population, while others may only serve one type of patient population.

#### General population

Catering to a general population – or a non-specific patient population – means that the CH is able to attend to the patient regardless of their age and the municipality they come from. CHs that cater to a general population include the United States, United Arab Emirates, France, Brazil, Israel, Switzerland, Portugal, Spain, and Malta.

#### Rural population

There are CHs that cater to rural populations as opposed to patients of a specific demographic profile (16). These CHs operate in remote settings and provide emergency, general, and surgical care to a local population situated near the hospital (17). Countries with rural CHs include the United States, Canada, Japan, the United Kingdom, Germany, Norway, Sweden, Australia, and New Zealand.

#### Older adult population

Countries with CHs that cater to the older adult are Japan, Singapore, Taiwan, Korea, the UK, Netherlands, Italy, Sweden, and Australia.

CHs that cater to the older adult population tend to provide post-acute care, such as rehabilitation services to reduce prolonged acute hospital stays and decrease hospital readmission rates among the older population that typically requires a longer recovery period (18).

Several CHs were observed to provide COVID-19 services to cope with the pandemic. Examples include COVID-19 testing sites (19–21), providing COVID-19 vaccinations (19–21), converting wards into COVID-19 wards (22–29), and converting hospitals into COVID-19 facilities (30–33). These countries include the United States, Canada, Japan, Singapore, Taiwan, Korea, the United Arab Emirates, Israel, the UK, Netherlands, Italy, Spain, Germany, Portugal, Australia, and New Zealand.

### State of research in CH settings focusing on post-acute care

Out of 470 studies, 40 studies (8.5%) were from countries with CH models focusing on post-acute care. Table 4 depicts the number of studies conducted in these countries from 2016 to 2024. An average of four articles were published each year and a maximum of 10 articles were published in 2021. Five studies (12.5%) employed primarily qualitative approaches. More than half (*n* = 28, 70%) employed a quantitative study approach, and seven studies adopted a mixed methods design (17.5%).

**Table 4:** Characteristics of studies included for examining research areas explored in high-income countries’ community hospitals with post-acute care focus (*n* = 40).

Variables	*n* (%)
Country of study	
Italy	2 (5.0)
Malta	1 (2.5)
Netherlands	2 (5.0)
Singapore	20 (50)
Spain	1 (2.5)
Sweden	2 (5.0)
United Kingdom	11 (27.5)
Japan	1 (2.5)
Study design	
Mixed methods	7 (17.5)
Quantitative	28 (70)
Qualitative	5 (12.5)
Year of study	
2016	1 (2.5)
2017	3 (7.5)
2018	2 (5.0)
2019	3 (7.5)
2020	2 (5.0)
2021	10 (25)
2022	8 (20)
2023	8 (20)
2024	3 (7.5)

Most studies were conducted in Singapore, an Asian country (*n* = 20, 43.3%). The rest were conducted in European countries (*n* = 19, 56.7%), where the majority were conducted in UK (*n* = 11, 58.8%) (Table 4).

### Key thematic categories on the state of research

**Figure 3:**
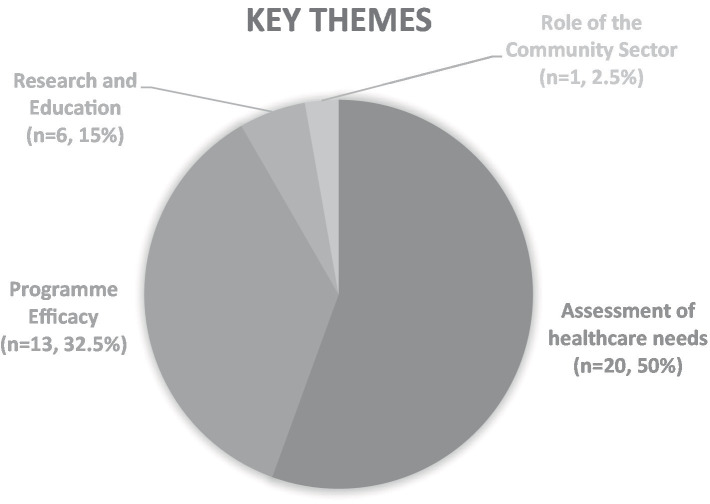
Key themes of the state of research in community hospitals.

The types of research in articles (*n* = 40) can be categorized into five broad categories (Figure 3)—assessment of healthcare needs, program efficacy, research and education, review of evidence, and role of the community sector.

#### Assessment of healthcare needs

Majority focused on assessing patients’ healthcare needs (*n* = 20, 50%). Five studies conducted in Singapore centered on obtaining an in-depth understanding of a specific condition to assess program effectiveness (34–38). This included investigating the factors linked to frailty within a subacute geriatric unit (34); assessing the correlation between factors related to acute hospital admissions and the enhancement of functionality upon discharge from a CH (35); assessing the correlation between nutritional status and rehabilitation effectiveness for postoperative hip fracture patients (36); uncovering the link between heightened psychological resilience and enhanced functional outcomes among post-operative hip fracture patients (37); and investigating the elements related to sarcopenia among older adults (38).

Two studies evaluated the characteristics of hospitalized patients and whether their healthcare needs were met (39, 40). One study sought to understand how patient characteristics and the factors linked to mortality a year after being admitted to an intermediate care unit would influence priorities for care in England (39); Another study envisioned optimal care for patients via a clinical characterization of patients from rural CHs in Sweden (40).

Three studies investigated patients’ experiences of being in a CH (41–43). One study studied the role of CHs via patients’ experiences in the UK (41); Another study aimed to understand patient experience and satisfaction from staying in an acute geriatric CH in Netherlands (42), and the last study investigated first-hand accounts of stroke experiences in Singapore (43).

Four studies focused on factors associated with the COVID-19 pandemic (44–47). One study assessed vaccine hesitancy levels among CH staff in Singapore to facilitate strategies to increase vaccine acceptance (44). Two studies investigated the ramifications of pandemic measures in Singapore qualitatively (45), and quantitatively (46); the last paper studied levels of loneliness among patients amidst COVID-19 restrictions in Malta (47).

Four studies utilized a methodological approach, focusing on critiquing the efficacy and significance of CHs in the healthcare landscape in England (18, 48, 49), and Europe (50).

Lastly, two studies identified patient sub-groups at greater risk of experiencing particular outcomes (51, 52). One study in Italy identified sub-groups that could benefit the most from CH care (51); the other study studied the impact of social support on rehabilitation outcomes among older adults that have undergone hip fracture surgery in Singapore (52).

#### Program efficacy

The next theme was on program efficacy (*n* = 13, 32.5%). Firstly, five of these articles centered on the efficacy of specific interventions in the CH setting (53–57). One study conducted in Singapore sought to ascertain if the Fatigue, Resistance, Ambulation, Illness, and Loss of weight scale is linked to patient rehabilitation outcomes (53); another study in Sweden studied the impact of oral neuromuscular training among older individuals experiencing swallowing difficulties (54); the third study assessed the outcomes of electromechanical gait trainers when used with conventional physiotherapy in Singapore (55); the fourth study explored the effectiveness of deprescribing rounds in Singapore (56); and the fifth study aimed to validate the modified version of the patient-reported experience measure in Japan (57).

Secondly, five studies examined the impact of the care model on patient outcomes (58–62), such as validating a tool to examine the degree of inter-professional collaboration in a CH in Singapore (58); examining if Municipal Acute Wards impacted admissions to general hospitals in Norway (59); determining the relationship between 12 h shifts for nurses and the rate of CH patient incidents in England (60); understanding how CHs meet the needs of older patients in Italy (61); and revamping care provision according to evolving care requirements of CH patients in Singapore (62).

Thirdly, one study in the UK evaluated the effectiveness of the care model by exploring stakeholders’ perceptions on intensive community support for admissions to CHs (63).

Lastly, two articles compared care experiences across settings (64, 65). One study in the UK investigated performance disparities among CHs by assessing relative cost efficiency, impact, and factors enhancing the effectiveness of inpatient rehabilitation for older individuals (64); another study in Netherlands contrasted care delivery for older patients in the acute geriatric CH versus a hospital setting (65).

#### Research and education

Six articles (15%) focused on research and educational activities within a CH setting (66–71). One study discussed factors linked to osteoporosis awareness among women patients in Singapore (66); another study in the UK focused on the responses of small, rural CHs to the COVID-19 pandemic (67); a study in Singapore assessed how caregivers’ dispositions and accessibility affect rehabilitation outcomes (68); another study examined factors impacting medication adherence among individuals with chronic illnesses in Singapore (69); the fifth study devised a scoring mechanism to forecast the mortality of patients in a subacute geriatric unit in Singapore (70); the last study documented distress levels among CH physicians, nurses, and rehabilitation therapists in Singapore (71).

#### Role of the community sector

One article (2.5%) examined the role and extent of community support for CHs in the UK (72).

#### Common limitations

Common limitations cited in the studies are the lack of generalizability as studies were conducted at a single site (36, 38, 57, 58, 69, 71), small sample sizes (34, 42, 43, 46, 51, 63, 66, 70, 71), or being conducted on a specific demographic profile that may not be representative of all service users (35, 36, 38, 39, 42, 43, 45, 46, 52, 54, 57, 62, 63, 66–71).

## Discussion

### CH models of care

Our study expanded the scope of a previous scoping review (5) to include CHs in high-income countries and uncovered CH models in rural and urban settings. Many CHs adapted to the COVID-19 pandemic by providing COVID-19 healthcare services. Furthermore, the challenges associated with an ageing population in Asian countries have prompted policymakers to plan future healthcare policies accordingly (73). Therefore, it is evident that CHs can evolve to cater to the needs of their healthcare landscape.

### State of research in CHs focusing on post-acute care

To our knowledge, this is the first study that examines the types of research conducted in CHs providing post-acute care. There is a paucity of research conducted in CHs due to clinical tasks taking precedence over research activities (74); few experienced researchers (74), and academic institutions being given priority in receiving research funds (74, 75).

Most studies centered on the assessment of healthcare needs, which includes the evaluation of characteristics of hospitalized patients, studying patients’ experiences of being in a CH, assessing the impact of COVID-19 on patient outcomes, and examining the efficacy of CHs. This highlights the importance of assessing the needs of CH patients as it informs the direction of the care model which would impact patient outcomes.

Potential research inquiries could consider themes related to systematic reviews of clinical guidelines, treatments, or practices in CH settings; research and educational needs among staff; role of the community in supporting the post-acute sector; and topics that have been under-examined in the existing literature. With post-acute services playing a significant role in referring patients to the appropriate community services, insights into the types of community support and barriers to service provision can identify areas for collaboration.

### Limitations and strengths of study

Our scoping review has several limitations. Firstly, the quality of evidence and efficacy of the different CH models was not evaluated. Secondly, these findings may not be transferable to non-high-income countries. Thirdly, our aim to understand the state of research meant focusing on the studies’ research objectives, rather than their findings.

Nevertheless, the strength of our scoping review lies in drawing upon a heterogeneous pool of studies to provide a snapshot of the current CH models in high-income countries.

## Conclusion

This scoping review demarcated current care models of CHs in high-income countries, with some focusing on acute care, sub-acute care, or a combination of both. CHs may also cater to more general patient populations or specific sub-groups. With the post-acute sector playing an increasingly significant role in meeting the healthcare needs of the population, policymakers can draw on the differing care models in this paper to identify potential adjustments to current services. An overview of the state of research is also pertinent to building the knowledge base necessary to support the development of policies in the post-acute sector.

## Data Availability

The original contributions presented in the study are included in the article/supplementary material, further inquiries can be directed to the corresponding author.
